# *In Silico* Analysis of Usher Encoding Genes in *Klebsiella pneumoniae* and Characterization of Their Role in Adhesion and Colonization

**DOI:** 10.1371/journal.pone.0116215

**Published:** 2015-03-09

**Authors:** Fida Khater, Damien Balestrino, Nicolas Charbonnel, Jean François Dufayard, Sylvain Brisse, Christiane Forestier

**Affiliations:** 1 LMGE—UMR CNRS 6023- Clermont Ferrand, 63000, France; 2 CIRAD, UMR 1334 AGAP, Avenue Agropolis, 34398 Montpellier, France; 3 Institut Pasteur, Microbial Evolutionary Genomics, 75015 Paris, France; 4 CNRS, UMR 3525, Paris, France; Quuen's University Belfast, UNITED KINGDOM

## Abstract

Chaperone/usher (CU) assembly pathway is used by a wide range of *Enterobacteriaceae* to assemble adhesive surface structures called pili or fimbriae that play a role in bacteria-host cell interactions. *In silico* analysis revealed that the genome of *Klebsiella pneumoniae* LM21 harbors eight chromosomal CU loci belonging to γκп and ϭ clusters. Of these, only two correspond to previously described operons, namely type 1 and type 3-encoding operons. Isogenic usher deletion mutants of *K*. *pneumoniae* LM21 were constructed for each locus and their role in adhesion to animal (Intestine 407) and plant (*Arabidopsis thaliana*) cells, biofilm formation and murine intestinal colonization was investigated. Type 3 pili usher deleted mutant was impaired in all assays, whereas type 1 pili usher deleted mutant only showed attenuation in adhesion to plant cells and in intestinal colonization. The LM21Δ*kpjC* mutant was impaired in its capacity to adhere to *Arabidopsis* cells and to colonize the murine intestine, either alone or in co-inoculation experiments. Deletion of LM21*kpgC* induced a significant decrease in biofilm formation, in adhesion to animal cells and in colonization of the mice intestine. The LM21∆*kpaC* and LM21∆*kpeC* mutants were only attenuated in biofilm formation and the adhesion abilities to *Arabidopsis* cells, respectively. No clear *in vitro* or *in vivo* effect was observed for LM21∆*kpbC* and LM21∆*kpdC* mutants. The multiplicity of CU loci in *K*. *pneumoniae* genome and their specific adhesion pattern probably reflect the ability of the bacteria to adhere to different substrates in its diverse ecological niches.

## Introduction

Bacterial adhesion to epithelial cells and abiotic surfaces is frequently mediated by diverse surface proteinaceous appendages referred to as adhesins. Gram-negative bacteria develop several fimbrial adhesins that are proteinaceous non-flagellar filaments with thin hair-like extension on the bacterial cell surface [1]. They are assembled by dedicated secretion systems and are composed primarily of the major repeating subunit protein with minor-subunit proteins including the adhesion subunits that enable the bacteria to specifically target a cell component or a surface [2]. Studies on the biochemistry and genetics of fimbrial biosynthesis have given rise to a nomenclature that distinguishes between fimbriae on the basis of their assembly mechanism [3]. The chaperone/usher (CU)-dependent pathway represents the most abundant secretory pathway among Gram-negative bacteria [3]. It is composed of a chaperone periplasmic protein and an outer membrane protein named the usher. The chaperone prevents self-aggregation of the fimbriae subunits and directs them to the usher, which in turn is involved in the secretion and correct assembly of the external fimbrial subunits [3]. Genes encoding the CU pathway are located on both chromosomal and plasmid molecules and are clustered with similar organization among different bacteria: an upstream region containing regulatory genes and a single downstream operon containing the required structural and assembly components. In addition, multiple CU pathways may be present in a single bacterial genome, which presumably confers the ability to adhere to a variety of different receptors and surfaces [4–6]. Expression of the CU gene clusters is typically highly regulated, subject to phase variation and responsive to environmental cues [7]. This cross-talk probably ensures that each bacterium does not express all pilus types at a given time, enabling the control of adhesive specificity.

The CU family has been described among members of the beta-proteobacteria and in cyanobacteria [3,8], but is mostly prevalent among enteric members of the γ-proteobacteria, including *Escherichia coli*, *Salmonella* and *Yersinia*. *Klebsiella pneumoniae* is a pathogenic *Enterobacteriaceae* that commonly causes nosocomial, increasingly multidrug resistant infections and is also emerging as a community pathogen [2,9,10]. Two classical CU fimbriae have been described in *Klebsiella*, type 1 and type 3-pili. By binding to mannosylated glycoproteins, type 1 pili allow bacterial adhesion to uroepithelial cells and the development of cystitis [11–13]. Type 3 fimbriae mediate adhesion to several cell types *in vitro* such as tracheal epithelial cells, renal tubular cells, extracellular matrix proteins and the membrane of human lung tissue [14–16]. In addition, both fimbriae have been associated with biofilm formation in *K*. *pneumoniae* [2,17]. Recently Wu et al. [18] described nine fimbrial loci found in *K*. *pneumoniae* NTUH- K2044: *fim*, *mrk*, and seven other fimbrial loci called *kpa* to *kpg*. Apart from the NTUH-K2044 *kpc* locus, no studies have investigated the *in vitro* and/or *in vivo* role of the other *K*. *pneumoniae* accessory fimbrial potential operons [18].

Given the recent increase in *K*. *pneumoniae* clinical importance and its well recognized diversity of niches, we assessed the presence of CU-like genes in this pathogen by searching for genes encoding putative fimbrial usher proteins. We analyzed their distribution, genetic conservation and genetic location. We used a reverse genetics approach to further investigate adhesion and colonization phenotype associated with eight LM21 usher operon candidates and *in vitro* and *in vivo* models.

## Material and Methods

### Identification of Chaperone-Usher loci in *K*. *pneumoniae* LM21

*K*. *pneumoniae LM21* was isolated from a cutaneous wound of a patient hospitalized in a intensive care unit of the teaching hospital of Clermont-Ferrand. Its sequencing was performed by GATC Biotech (Konstanz, Germany) using Illumina technology and a 2×100 nucleotide (nt) paired-end strategy. All reads were pre-processed to remove low quality or artefactual nucleotides. First, all nucleotides occurring at 5' and 3' ends and supported by a Phred quality score < 30 were trimmed off using Sickle (http://www.github.com/najoshi/sickle). Second, contaminant oligonucleotides (i.e. library adaptors) were detected and trimmed off using AlienTrimmer [19]. Third, reads shorter than 60 nt after the above cleaning steps were discarded, as well as those containing more than 20% nucleotides with Phred score < 30. Resulting reads were assembled using clc_assembler from the CLC Genomics Workbench analysis package (http://www.clcbio.com/products/clc-genomics-workbench/). Contigs were reordered and reoriented, using as reference the genomic sequence of strain MGH 78578, with Mauve Contig Mover [20]. Obvious contaminant contigs were discarded. 154 remaining contigs (total genome size: 5,509,751 nt; N50, 104,592 nt) were subsequently imported into the Microscope database system [21]. The genome was annotated automatically within the MicroScope platform and manually visualized using the Magnifying Genomes (MaGe) web interface [22,23]. The LM21 genome sequence was deposited in the EMBL database and is available from the INSDC databases (Assembly_name: KPLM21; Study_ID: PRJEB7075; Sample_ID: ERS537769 | Contigs: CCVM01000001-CCVM01000154).

The NCBI Blast 2.28+ program was used to look for the presence of usher-like sequences in *K*. *pneumoniae* strain LM21. Usher amino acid sequences annotated in NCBI were used as initial BLASTp queries to look for the presence of CU in *K*. *pneumoniae* LM21. BLASTp searches were performed using the BLOSUM62 matrix and an E-value cut-off score of 0.1. Newly identified proteins with a reported E-value of 0 were retained, whereas hits with an E-value >0 were screened for the presence of an usher protein family domain (PFAM00577) and/or flanking chaperone (PF00345, PF02753 or COG3121) for encoding genes before they were added to the usher query list. The NCBI Conserved Domain Database (CDD) component of the NCBI Entrez query retrieval system was used to examine amino-acid sequences for conserved domain [24].

After each BLASTp run, the updated usher query database was used to re-probe the genome sequences until no new sequences were found. All putative usher amino-acid sequences encoded by LM21 *K*. *pneumoniae* genome were downloaded from MaGe database. They are listed in S1 Table. The presence of the eight usher encoded genes predicted by *in silico* analysis was verified by PCRs using primers listed in Table 1 (P1 to P16).

**Table 1:** Primers used in this study.

*Num*	*Primers Name*	Oligonucleotide sequence (5'- 3')	PCR product size (bp)	Use
P1	LM21_*kpaC*-Fw	CCGGGCACCTATCACCTG	607 pb	Primers used to check the presence of usher LM21*kpaC*
P2	LM21_*kpaC*-Rv	GCTTAACCGGCACGCTGG		
P3	LM21_*kpbC*-Fw	GTCCTGGCGAGTTCGTTACGG	658 pb	Primers used to check the presence of usher LM21*kpbC*
P4	LM21_*kpbC*-Rv	CGTTGGCGCCGTAGTCGATACG		
P5	LM21_*kpd*C-Fw	GAGATCGCCTGCAGCGGCAG	568 pb	Primers used to check the presence of usher LM21*kpdC*
P6	LM21_*kpdC*-Rv	CAGCGCTGTCCACCGTCGC		
P7	LM21_*kpeC*-Fw	CCGCCAACAGCGATGATAAGCC	509 pb	Primers used to check the presence of usher LM21*kpeC*
P8	LM21_*kpeC*-Rv	GAACCCGCGCATCAGCGAC		
P9	LM21_*kpgC*-Fw	GGTATTGACCGGCAGCGTG	574 pb	Primers used to check the presence of usher LM21*kpgC*
P10	LM21_*kpgC*-Rv	GCGGGCAAAGCATCGACG		
P11	LM21_*kpj*C-Fw	TCTCTTTGCGAAGCCAAC	582 pb	Primers used to check the presence of usher LM21*kpjC*
P12	LM21_*kpj*C-Rv	CATGGATCGGGAATGGAT		
P13	LM21_*mrkC*-Fw	GCCGCAAAGCGGTTGGTG	592 pb	Primers used to check the presence of usher LM21*mrkC*
P14	LM21_*mrkC-*Rv	CGACGCCGTTCTACATTACC		
P15	LM21_*fimC*-Fw	GCTGACCCCGTAATAGAG	475 pb	Primers used to check the presence of usher LM21*fimC*
P16	LM21_*fimC*-Rv	CGCGCCACCCTCTATCTC		
P17	LM21_*kpa-Kana-*Fw	AGCCATGACGCAGGCGCGACTGCTTCTCCCTGCCTGTCCGCGGAGTATCTGAATTCATGGGTAGGCTGGAGCTGCTTCG	1595 pb	LM21Δ*kpa* isogenic mutant construction
P18	LM21_*kpa-Kana-*Rv	CATATCAGCCCCTCTTCCATGTTGAAAACCTGCATCCATTACCGACATTCGGCCTGATAGCATATGAATATCCTCCTTAGTTC		
P19	LM21_*kpb-Kana*-Fw	AGCATAGTAGGGATAAAAAAAGAAGTATTTTTCTCATTCTTTATTGTTCCTTGGTCAATAGTAGGCTGGAGCTGCTTCG	1595 pb	LM21Δ*kpb* isogenic mutant construction
P20	LM21_*kpb-Kana-Rv*	GATGTGTCTATGCCAAATAAGAATAATGGATTATTCAAACTATCCACCATCTTCTTTGCACATATGAATATCCTCCTTAGTTC		
P21	LM21_*kpd-Kana-*Fw	TCAGGGGTACCTCTTCTGCTGGCCGGCGGGTACTGCGCTTCGCTGTCGGCAGCGTGCCAGGTAGGCTGGAGCTGCTTCG	1595 pb	LM21Δ*kpd* isogenic mutant construction
P22	LM21_*kpd-Kana-*Rv	ACAGGAAGTTGATGTTTCCTTTACTGGCGCATGCCTGACGGCGCTGTGCCGCCAGGCAGACATATGAATATCCTCCTTAGTTC		
P23	LM21_*kpe-Kana-*Fw	GCGTCAGGGCGACCCGACGCAGGAGAGTTAAAAAGGACATGATAACGTCTGCTGTAAGATGTAGGCTGGAGCTGCTTCG	1595 pb	LM21Δ*kpe* isogenic mutant construction
P24	LM21_*kpe-Kana-*Rv	TTTGGCGCTGTGCAGACGTTTAGCGCCCCCGTTTCGGCGCACTAAGCGTCTGATGTCGCGCATATGAATATCCTCCTTAGTTC		
P25	LM21_*kpg-Kana-*Fw	GCGCTCAGCCCCAGCAGAAAGAGGAAAGGAAGTAGACGAAAAACAGATTTCATAGCCGCCGTAGGCTGGAGCTGCTTCG	1595 pb	LM21Δ*kpg* isogenic mutant construction
P26	LM21_*kpg-Kana-*Rv	AGAATAACAACAGCCAGGGTGCGGGAGTATGCCTTGTTGCCCGTTACCGCCGGGCGGGCGCATATGAATATCCTCCTTAGTTC		
P27	LM21_*kpj-Kana*-Fw	ATTAATGGGTATGGAATTTTGGGTCAGTTCAATTTCAATATGAAATATGCATGCCTCTTGGTAGGCTGGAGCTGCTTCG	1595 pb	LM21Δ*kpj* isogenic mutant construction
P28	LM21_*kpj-Kana-*Rv	ACCACTTCATTTTATTCTTCCTCGTAAGGACGGCTATTTACTTTGCTCAAGGGCGCAGAGCATATGAATATCCTCCTTAGTTC		
P29	LM21_*mrk-Kana-*Fw	ATTAAGACGATAAAAAGCGTTAGTAATTTCCTCAGCGACATACGCTATCCTTTGTTGTTTGTAGGCTGGAGCTGCTTCG	1595 pb	LM21Δ*mrk* isogenic mutant construction
P30	LM21_*mrk-Kana-*Rv	CATTAATGACTATGGCGCCGATGTCGCAGTGAAAGTGGCCGTTAAATAAGGGAAAAGCTACATATGAATATCCTCCTTAGTTC		
P31	LM21_*fim-Kana-*Fw	GGGACACGCTGCGCATGACCAGAAGCAATGACAGCCGGGCAACGTCGGACCGGAGGGATAGTAGGCTGGAGCTGCTTCG	1595 pb	LM21Δ*fim* isogenic mutant construction
P32	LM21_*fim-Kana-*Rv	GGTGCGCCCAGGGTGAACAGGGCGCCCAGCAGATACTGCAGTGTTCTCATCATGATCTCCCATATGAATATCCTCCTTAGTTC		
P33	*kana-*Fw	GTAGGCTGGAGCTGCTTCG	1475 pb	Primers used to check the presence of kanamycin cassette
P34	*kana-*Rv	CATATGAATATCCTCCTTAGTTC		
P35	LM21_*kpa*-Fw	GTACGCCAACGTCTTCCGC	2451 pb	Two-step PCR amplification to generate fragment containing *kpa* operon promoter fused to LM21*kpaC*
P36	LM21_P_*kpa*-Rv	TTCCCTGACATACCATAATCATCCTGGCCGCGTTCGATAC		
P37	LM21_P_*kpa-*Fw	GTATCGAACGCGGCCAGGATGATTATGGTATGTCAGGGAA		
P38	LM21_*kpa*-Rv	TTACCGACATTCGGCCTG		
P39	LM21_*kpb*-Fw	GGTGAAAAAGCATCTGAGC	2661 bp	Two-step PCR amplification to generate fragment containing *kpb* operon promoter fused to LM21*kpbC*
P40	LM21_P_*kpb*-Rv	CCATTATTCTTATTTGGCATCTACCGGTTTAGCACGACC		
P41	LM21_P_*kpb-*Fw	GGTCGTGCTAAACCGGTAGATGCCAAATAAGAATAATGG		
P42	LM21_*kpb*-Rv	TTAGCGACAAACCGTTTCG		
P43	LM21_*kpd*-Fw	GAAAGCGCAGGGAGTATG	2454 bp	Two-step PCR amplification to generate fragment containing *kpd* operon promoter fused to LM21*kpdC*
P44	LM21_P_*kpd*-Rv	CTTGATCTCCCTGAGTTTCATTCAACATAATGACCTCCTGGC		
P45	LM21_P_*kpd-*Fw	GCCAGGAGGTCATTATGTTGAATGAAACTCAGGGAGATCAAG		
P46	LM21_*kpd*-Rv	TTACTTCTCCTTTATTCCCGG		
P47	LM21_*kpe*-Fw	CTATGATGAATCCTATGCCG	2762 bp	Two-step PCR amplification to generate fragment containing *kpe* operon promoter fused to LM21*kpe*C
P48	LM21_P_*kpe*-Rv	CCGGGCCGTGGCGCATGATCGAGCTGCTTTTTCAGC		
P49	LM21_P_*kpe-*Fw	GCTGAAAAAGCAGCTCGATCATGCGCCACGGCCCGG		
P50	LM21_*kpe-*Rv	TTAAAAAGGACATGATAACGTC		
P51	LM21_*kpg*-Fw	CTAATATCCTGCGGAAGG	2802 pb	Two-step PCR amplification to generate fragment containing *kpg* operon promoter fused to LM21*kpgC*
P52	LM21_P_*kpg*-Rv	CGCCGTCCGGTAAGCTCATTCAGGATTGATAGTGTTGGC		
P53	LM21_P_*kpg-*Fw	GCCAACACTATCAATCCTGAATGAGCTTACCGGACGGCG		
P54	LM21_*kpg*-Rv	TCAGCGTGTCTGGCACAG		
P55	LM21_*kpj*-Fw	GCTTTAACTCGCAGAAAGG	2927pb	Two-step PCR amplification to generate fragment containing *kpj* operon promoter fused to LM21*kpjC*
P56	LM21_P_*kpj*-Rv	AATCCATCGTTGAGGCATTCAGCTGGTCCAGGTGG		
P57	LM21_P_*kpj*-Fw	CCACCTGGACCAGCTGAATGCCTCAACGATGGATT		
P58	LM21_*kpj*-Rv	TTAACTTTCATTCTCTTCTCCTGAC		
P59	LM21_*mrk*-Fw	GCTGACCAACAAAATCATCC	2794 pb	Two-step PCR amplification to generate fragment containing *mrk* operon promoter fused to LM21*mrkC*
P60	LM21_P_*mrk*-Rv	CAGAATGACCTCTGCTTCATGCTGACCTCAGAATAAAAATACA		
P61	LM21_P_*mrk*-Fw	TGTATTTTTATTCTGAGGTCAGCATGAAGCAGAGGTCATTCTG		
P62	LM21_*mrk*-Rv	GCCATTAAGACGATAAAAAGC		
P63	LM21_*fim*-Fw	CAAATTTTATGTTAACGACGC	2893 pb	Two-step PCR amplification to generate fragment containing *fim* operon promoter fused to LM21*fim*C
P64	LM21_P_*fim*-Rv	CCATATTTCCGATGTGACATTCATGCGCGAATATCGTC		
P65	LM21_P_*fim-*Fw	GACGATATTCGCGCATGAATGTCACATCGGAAATATGG		
P66	LM21_*fim*-Rv	GACGTTATCATGTCCTTTTTAA		
P67	LM21-*kpa-*RT-qPCR-Fw	GCTCCGACGATAAAGAATATAAC	113 pb	Primers used for qPCR to verify the expression of *kpaC*
P68	LM21_*kpa-*RT-qPCR-Rv	CAGGTTGGTGTATTTATCTTCC		
P69	LM21_*kpb-*RT-qPCR-Fw	GGTAATTATACCTCTCGCTCAC	114 pb	Primers used for qPCR to verify the expression of *kpbC*
P70	LM21_*kpb-*RT-qPCR-Rv	GTCCCAGTAATCTTCAATCAAC		
P71	LM21_*kpd-*RT-qPCR-Fw	CTGTCATTTAACGGAATCTGG	112 pb	Primers used for qPCR to verify the expression of *kpdC*
P72	LM21_*kpd-*RT-qPCR-Rv	GCTGGGCATACAGATAATAATTG		
P73	LM21_*kpe-*RT-qPCR-Fw	CAATACCATAACCTCTCGGTC	149 pb	Primers used for qPCR to verify the expression of *kpeC*
P74	LM21_*kpe-*RT-qPCR-Rv	CGATCAAAACCTTTGCTGTAC		
P75	LM21_*kpg-*RT-qPCR-Fw	GATGTCTGACGATGAAATGC	125 pb	Primers used for qPCR to verify the expression of *kpgC*
P76	LM21_*kpg-*RT-qPCR-Rv	CAAAGGTCTCGTAGATCACATAC		
P77	LM21_*kpj-*RT-qPCR-Fw	CTGATATCACGTTTGTTGACG	121 pb	Primers used for qPCR to verify the expression of *kpj*C
P78	LM21_*kpj-*RT-qPCR-Rv	CGGGAATAAACTTCTCGATC		
P79	LM21_*mrk-*RT-qPCR-Fw	CTGTGGTTTGGCGATAAC	125 pb	Primers used for qPCR to verify the expression of *mrkC*
P80	LM21_*mrk-*RT-qPCR-Rv	CCGTAGTTAAGGTTGTTTTCAC		
P81	LM21_*fim-*RT-qPCR-Fw	GAAAAACATGGGCTATTTCG	115 pb	Primers used for qPCR to verify the expression of *fimC*
P82	LM21_*fim-*RT-qPCR-Rv	GGATTTGTTATAGAGAAAACGC		
P83	RT-*gyrA*-Fw	ATTGGGCAATGACTGGAACAA	149 pb	Primers used for qPCR to verify the expression of *gyrA* in LM21 wild type
P84	RT-*gyrA*-Rv	CCACCAGCATGTAACGCAG		

### Locus structure prediction and genetic analysis

To define potential operon genetic organization, we visualized flanking regions of usher nucleotide sequences in xBase2 [25]. Fimbrial encoding genes were identified using conserved protein domain searches [24] and sequence homology to annotated genes. Intergenic regions >200pb were investigated for the presence of protein encoding sequences with conserved fimbrial domains or significant sequence identity to fimbrial subunits.

### Multiple sequence alignment and phylogenetics

Publicly available *K*. *pneumoniae* genomes used for this study were those from strains HS11286, MGH 78578, NTUH-K2044, 342 (reassigned to *K*. *variicola* since genome sequencing), KCTC 2242, 1084, JM45, CG43, Kp13, 30684/NJST258_2 and 30660/NJST258_1.

Full-length usher amino acid sequences from intact fimbrial operons were used to infer evolutionary relationships. Sequences were aligned in MAFFT v6.617b [26,27], using iterative global-pair refinement method with default gap penalties. The alignment was cleaned with trimal [28] so that every site with more than 30% of gaps or with an average similarity inferior to 0.0001 was removed.

Phylogenetic analyses were performed with PhyML 3.1 [29], using LG+gamma model with a 4 categories gamma law. To estimate the confidence in the tree topology, statistical aLRT-SH-like supports were computed [30]. Alignments and phylogenetic tree were repeated with usher sequences of previous usher phylograms [3] to check tree validity (data not shown).

### Bacterial strains, plasmids and growth conditions

The bacterial strains and plasmids used in the study are shown in Table 2. All bacterial strains were stored at −80°C in Lysogeny Broth (LB) medium containing 20% glycerol. When appropriate, antibiotics were added to the media at the following concentrations: ampicillin (50μg/ml), kanamycin (50μg/ml), tetracycline (20μg/ml), streptomycin (50μg/ml) and spectinomycin (50μg/ml). LB media, 0.4% glucose M63B1 minimal medium (M63B1–0.4% Glu) and Dulbecco’s modified Eagle’s medium (DMEM) were used for experiments. Bacterial growth was monitored by measuring the optical density at 620 nm (OD_620_) and plating dilution on agar plates to determine colony forming units.

**Table 2:** Strains and plasmids used.

Num	Strains or Plasmids	Decription	Source and/or reference
S1	LM21 *gfp*	LM21 strain SHV-1::*aadA7*-*gfp*mut3; serogroup O25, K35, Sp^r^	[64]
S2	LM21 *gfp*/pSTAB	LM21 gfp/pSTAB; Sp^r^, Ap^r^	This study
S3	LM21 *gfp*-*Strepto*/pSTAB	LM21 *gfp*/pSTAB; Sp^r^, Ap^r^, St^r^	This study
S4	LM21Δ*kpaC*-*kana*	LM21 *gfp* Δ*kpaC*::KmFRT; Sp^r^, Km^r^	This study
S5	LM21Δ*kpbC*-*kana*	LM21 *gfp* Δ*kpbC*::KmFRT; Sp^r^, Km^r^	This study
S6	LM21Δ*kpdC*-*kana*	LM21 *gfp* Δ*kpdC*::KmFRT; Sp^r^, Km^r^	This study
S7	LM21Δ*kpeC-kana*	LM21 *gfp* Δ*kpeC*::KmFRT; Sp^r^, Km^r^	This study
S8	LM21Δk*pgC*-*kana*	LM21 *gfp* Δ*kpgC*::KmFRT; Sp^r^, Km^r^	This study
S9	LM21Δ*kpjC*-*kana*	LM21 *gfp* Δ*kpjC*::KmFRT; Sp^r^, Km^r^	This study
S10	LM21Δ*mrkC*-*kana*	LM21 *gfp* Δ*mrk*C::KmFRT; Sp^r^, Km^r^	This study
S11	LM21Δ*fimC*-*kana*	LM21 *gfp* Δ*fim*C::KmFRT; Sp^r^, Km^r^	This study
S12	LM21Δ*kpaC-kana*/pSTAB-*kpaC*	LM21 *gfp* Δ*kpa*C::KmFRT/pSTAB_*kpaC*; Sp^r^, Ap^r^, Km^r^	This study
S13	LM21Δ*kpbC-kana*/pSTAB-*kpbC*	LM21 *gfp* Δ*kpbC*::KmFRT/pSTAB_*kpbC*; Sp^r^, Ap^r^, Km^r^	This study
S14	LM21Δ*kpdC-kana*/pSTAB-*kpdC*	LM21 *gfp* Δ*kpd*C::KmFRT/pSTAB_*kpdC*; Sp^r,^ Ap^r^, Km^r^	This study
S15	LM21Δ*kpeC-kana*/pSTAB-*kpeC*	LM21 *gfp* Δ*kpeC*::KmFRT/pSTAB_*kpeC;* Sp^r^, Ap^r^, Km^r^	This study
S16	LM21Δ*kpgC-kana*/pSTAB-*kpgC*	LM21 *gfp* Δ*kpgC*::KmFRT/pSTAB_*kpgC*; Sp^r^, Ap^r^, Km^r^	This study
S17	LM21Δ*kpjC-kana*/pSTAB-*kpjC*	LM21 *gfp* Δ*kpjC*::KmFRT/pSTAB_*kpjC*; Sp^r^, Ap^r^, Km^r^	This study
S18	LM21Δ*mrkC-kana*/pSTAB-*mrkC*	LM21 *gfp* Δ*mrkC*::KmFRT/pSTAB_*mrkC*; Sp^r^, Ap^r^, Km^r^	This study
S19	LM21Δ*fimC-kana*/pSTAB-*fim*	LM21 *gfp* Δ*fimC*::KmFRT/ pSTAB_*fim*C, Sp^r^, Ap^r^, Km^r^	This study
S20	LM21Δ*kpaC-kana/*pSTAB	LM21 *gfp* Δ*kpaC*::KmFRT/pSTAB; Sp^r^, Km^r^, Ap^r^, Km^r^	This study
S21	LM21Δ*kpbC-kana/*pSTAB	LM21 *gfp* Δ*kpbC*::KmFRT/pSTAB; Sp^r^, Km^r^, Ap^r^, Km^r^	This study
S22	LM21Δ*kpdC-kana/*pSTAB	LM21 *gfp* Δ*kpdC*::KmFRT/pSTAB; Sp^r^, Km^r^, Ap^r^, Km^r^	This study
S23	LM21Δ*kpeC*-*kana*/pSTAB	LM21 *gfp* Δ*kpeC*::KmFRT/pSTAB; Sp^r^, Km^r^, Ap^r^, Km^r^	This study
S24	LM21Δ*kpgC-kana/*pSTAB	LM21 *gfp* Δ*kpgC*::KmFRT/pSTAB; Sp^r^, Km^r^, Ap^r^, Km^r^	This study
S25	LM21Δ*kpjC-kana/*pSTAB	LM21 *gfp* Δ*kpjC*::KmFRT/pSTAB; Sp^r^, Km^r^, Ap^r^, Km^r^	This study
S26	LM21Δ*mrkC-kana/*pSTAB	LM21 *gfp* Δ*mrkC*::KmFRT/pSTAB; Sp^r^, Km^r^, Ap^r^, Km^r^	This study
S27	LM21Δ*fimC-kana/*pSTAB	LM21 *gfp* Δ*fimC*::KmFRT/pSTAB; Sp^r^, Km^r^, Ap^r^, Km^r^	This study
S28	LM21Δ*kpaC-kana*-*Strepto*/pSTAB-*kpaC*	LM21 *gfp* Δ*kpaC*::KmFRT/pSTAB_*kpaC*; Sp^r^, Km^r^,Ap^r^, St^r^	This study
S29	LM21Δ*kpbC-kana*-*Strepto*/pSTAB-*kpbC*	LM21 *gfp* Δ*kpbC*::*KmFRT*/pSTAB_*kpbC*; Sp^r^, Km^r^, Ap^r^, St^r^	This study
S30	LM21Δ*kpdC-kana*-*Strepto*/pSTAB-*kpdC*	LM21 *gfp* Δ*kpdC*::KmFRT/pSTAB_*kpdC*; Sp^r^, Km^r^, Ap^r^, St^r^	This study
S31	LM21Δ*kpeC-kana*-*Strepto*/pSTAB-*kpeC*	LM21 *gfp* Δ*kpeC*::*KmFRT*/pSTAB_*kpeC*; Sp^r^, Km^r^, Ap^r^, St^r^	This study
S32	LM21Δ*kpgC-kana*-*Strepto*/pSTAB-*kpgC*	LM21 *gfp* Δ*kpgC*::*KmFRT*/pSTAB_*kpgC*; Sp^r^, Km^r^, Ap^r^, St^r^	This study
S33	LM21Δ*kpjC-kana*-*Strepto*/pSTAB-*kpjC*	LM21 *gfp* Δ*kpjC*::*KmFRT*/pSTAB_*kpjC*; Sp^r^, Km^r^, Ap^r^, St^r^	This study
S34	LM21Δ*mrkC-kana*-*Strepto*/pSTAB-*mrkC*	LM21 *gfp* Δ*mrkC*::*KmFRT*/pSTAB_*mrkC*; Sp^r^, Km^r^, Ap^r^, St^r^	This study
S35	LM21Δ*fimC-kana*-*Strepto*/pSTAB-*fimC*	LM21 *gfp* Δ*fimC*::*KmFRT*/pSTAB_*fimC*; Sp^r^, Km^r^, Ap^r^, St^r^	This study
S36	LM21Δ*kpaC-kana*-*Strepto/*pSTAB	LM21 *gfp* Δ*kpaC*::KmFRT/pSTAB; Sp^r^, Km^r^, Ap^r^, St^r^	This study
S37	LM21Δ*kpbC-kana*-*Strepto/*pSTAB	LM21 *gfp* Δ*kpbC*::KmFRT/pSTAB; Sp^r^, Km^r^, Ap^r^, St^r^	This study
S38	LM21Δ*kpdC-kana*-*Strepto/*pSTAB	LM21 *gfp* Δ*kpdC*::KmFRT/pSTAB; Sp^r^, Km^r^, Ap^r^, St^r^	This study
S39	LM21Δ*kpeC-kana*-*Strepto*/pSTAB	LM21 *gfp* Δ*kpeC*::KmFRT/pSTAB; Sp^r^, Km^r^, Ap^r^, St^r^	This study
S40	LM21Δ*kpgC-kana*-*Strepto/*pSTAB	LM21 *gfp* Δ*kpgC*::KmFRT/pSTAB; Sp^r^, Km^r^, Ap^r^, St^r^	This study
S41	LM21Δ*kpjC-kana*-*Strepto/*pSTAB	LM21 *gfp* Δ*kpj*C::KmFRT/pSTAB; Sp^r^, Km^r^, Ap^r^, St^r^	This study
S42	LM21Δ*mrkC-kana*-*Strepto/*pSTAB	LM21 *gfp* Δ*mrkC*::KmFRT/pSTAB; Sp^r^, Km^r^, Ap^r^, St^r^	This study
S43	LM21Δ*fimC-kana*-*Strepto/*pSTAB	LM21 *gfp* Δ*fimC*::KmFRT/pSTAB; Sp^r^, Km^r^, Ap^r^, St^r^	This study
S44	LM21Δ*kpaC*	LM21 *gfp* Δ*kpaC*; Sp^r^	This study
S45	LM21Δ*kpbC*	LM21 *gfp* Δ*kpbC*; Sp^r^	This study
S46	LM21Δ*kpdC*	LM21 *gfp* Δ*kpdC;* Sp^r^	This study
S47	LM21Δ*kpe*C	LM21 *gfp* Δ*kpeC*, Sp^r^	This study
S48	LM21Δ*kpgC*	LM21 *gfp* Δ*kpgC;* Sp^r^	This study
S49	LM21Δ*kpjC*	LM21 *gfp* Δ*kpj*C; Sp^r^	This study
S50	LM21Δ*mrkC*	LM21 *gfp* Δ*mrkC;* Sp^r^	This study
S51	LM21Δ*fimC*	LM21 *gfp* Δ*fimC*, Sp^r^	This study
S52	LM21Δ*kpaC*/pSTAB-*kpaC*	LM21 *gfp* Δ*kpaC*/pSTAB_*kpaC*; Sp^r^, Ap^r^	This study
S53	LM21Δ*kpbC*/pSTAB-*kpbC*	LM21 *gfp* Δ*kpbC*/pSTAB_*kpbC*; Sp^r^, Ap^r^	This study
S54	LM21Δ*kpdC*/pSTAB-*kpdC*	LM21 *gfp* Δ*kpd*C/pSTAB_*kpdC*; Sp^r^, Ap^r^	This study
S55	LM21Δ*kpeC*/pSTAB-*kpeC*	LM21 *gfp* Δ*kpeC*/pSTAB_*kpeC*; Sp^r^, Ap^r^	This study
S56	LM21Δ*kpgC*/pSTAB-*kpgC*	LM21 *gfp* Δ*kpgC*/pSTAB_*kpgC*; Sp^r^, Ap^r^	This study
S57	LM21Δ*kpjC*/pSTAB-*kpjC*	LM21 *gfp* Δ*kpjC*/pSTAB_*kpjC*; Sp^r^, Ap^r^	This study
S58	LM21Δ*mrkC*/pSTAB-*mrkC*	LM21 *gfp* Δ*mrkC*/pSTAB_*mrkC*; Sp^r^, Ap^r^	This study
S59	LM21Δ*fimC*/pSTAB-*fimC*	LM21 *gfp* Δ*fimC*/pSTAB_*fimC*; Sp^r^, Ap^r^	This study
S60	LM21Δ*kpaC/*pSTAB	LM21 *gfp* Δ*kpaC*/pSTAB; Sp^r^, Ap^r^	This study
S61	LM21Δ*kpbC/*pSTAB	LM21 *gfp* Δ*kpbC*/pSTAB; Sp^r^, Ap^r^	This study
S62	LM21Δ*kpdC/*pSTAB	LM21 *gfp* Δ*kpdC*/pSTAB; Sp^r^, Ap^r^	This study
S63	LM21Δ*kpeC/*pSTAB	LM21 *gfp* Δ*kpeC*/pSTAB; Sp^r^, Ap^r^	This study
S64	LM21Δ*kpgC/*pSTAB	LM21 *gfp* Δ*kpgC*/pSTAB; Sp^r^, Ap^r^	This study
S65	LM21Δ*kpjC/*pSTAB	LM21 *gfp* Δ*kp*C/pSTAB; Sp^r^, Ap^r^	This study
S66	LM21Δ*mrkC*/pSTAB	LM21 *gfp* Δ*mrkC*/pSTAB; Sp^r^, Ap^r^	This study
S67	LM21Δ*fimC/*pSTAB	LM21 *gfp* Δ*fimC*/pSTAB; Sp^r^, Ap^r^	This study
S68	TOP10	*E*.*coli* from invitrogen	Invitrogen
**Plasmids**	** **		
V1	pKOBEG199	Plasmid encoding Lambda Red recombinase protein	[31]
V2	pKD4	Plasmid with FRT-flanked kanamycin-resistance cassette used for kanamycin casette amplification, Km^r^, Ap^r^, Km^r^,	[33]
V3	pBlunt	Used for PCR product cloning	Invitrogen
V4	pSTAB	pZE derivative plamsid. Contains the *flm* toxin-antitoxin system from F plasmid; Ap^r^	Gift from J.M. Ghigo
V5	pCP20	pCP20 carries the yeast recombinase gene (FLP, aka exo), chloramphenicol and ampicillin resistant gene and temperature sensitive replication. Ap^r^	[32]

Abbreviations: Ap, ampicillin; Km, kanamycin; St, streptomycin; Sp, spectinomycin

### Cloning of the *K*. *pneumoniae* LM21 usher-like genes, construction of isogenic and transcomplementation mutants

Primers were designed on the basis of *K*. *pneumoniae* LM21 genome sequence information. All primers used are listed in Table 1. Chromosomal DNA extraction was performed by NucleoSpin tissue kit (Macherey-Nagel) according to the manufacturer’s recommendations.

The usher-defective mutants were created by allelic exchange after replacement of the Usher encoding gene by the selectable kanamycin resistance gene according to Chaveroche et *al*. [31]. The kanamycin cassette flanked by 60-bp fragments, which correspond to the encoding upstream and downstream regions of the usher encoding gene, was generated using pKD4 plasmid as template and primers P17 to P32 (Table 1). They were electroporated in the *K*. *pneumoniae* LM21 strain harboring the lambda-red protein-encoding plasmid pKOBEG199 under the control of a promoter induced by l-arabinose. Mutants were selected onto LB agar containing kanamycin. The loss of the pKOBEG199 plasmid was then checked on LB containing tetracycline. The substitution of the encoding usher gene by the kanamycin cassette was further checked by PCR performed with primers P33 and P34 (Table 1). In a second step, the antibiotic resistant encoding gene was excised from the mutant’s genome using the pCP20 plasmid, a temperature-sensitive replication plasmid with thermal induction of flippase recombinase (FLP) synthesis [32], which gave rise to nonpolar mutants as previously described in Datsenko et *al*. [33]. For *in vivo* assays, spontaneous mutants of Δ*usher-kana* (designated LM21Δ*usher*-*kana* in Table 2) were selected for streptomycin resistance (designated LM21Δ*usher*-*kana-strepto* in Table 2)

PCRs were performed using a Biorad T100 Thermal cycler. Restriction enzymes, Phusion high-Fidelity DNA polymerase and TaKaRa LA Taq were purchased from New England Biolabs, Thermo Scientific and Takara Biotechnology Inc, respectively, and used according to the manufacturers’ recommendations.

For transcomplementation assays, fragments containing the entire *usher-like* genes and their own putative promoters, as detected by sequence analysis, were amplified from *K*. *pneumoniae* LM21 genomic DNA by an overlapping extension PCR using primers listed in Table 1 (P35 to P66). The resulting fragment was cloned using Zero Blunt PCR cloning kit (Invitrogen) and subcloned into the *Eco*R1 or *Bam*H1 digested pSTAB vector. Resulting recombinant vectors (pSTAB-*usher*) were then introduced by electroporation into the isogenic mutants. In parallel, the LM21 wild type strain and the isogenic mutants were transformed with the empty pSTAB plasmid vector (Table 2).

### RNA manipulations, real-time RT-PCR

Total RNA was extracted from bacteria grown in 3 different media conditions (LB, M63B1 and DMEM), using Trizol reagent (Invitrogen) according to the method described by Toledo-Araba et *al*. [34] after lysing bacteria in the Precellys tissue homogenizer (Bertin Technologie). Reverse transcription was performed with the iScript cDNA synthesis kit (Biorad) using 1 μg of RNA on the T100 Thermal Cycler (Biorad) according to the manufacturers’ recommendations, and quantification of cDNA levels was done using the SsoAdvanced Universal SYBR Green Supermix (Biorad) on a C1000 thermal Cycler- CFX96 (Biorad) with primers P67 to P82 (Table 1). As internal controls, the *rpoB* gene was amplified with primers P83/P84. Amplification of a single expected product was confirmed by melting curve analysis. The amplification efficiency (E) of the reactions for the target genes was determined and used to compare relative gene expression. The ratio = (E_sample_)^ΔCPsample^ / (E_reference_)^ΔCPreference^ was calculated (CP: crossing point).

### Biofilm assays

The ability of bacteria to form biofilm was assessed in both a static microtiter plate and a dynamic microfermentor biofilm model. All experiments were performed in biological and technical triplicates. For the microtiter experiment measuring the early biofilm formation capacity, 4.10^6^ CFU/mL of an overnight culture were inoculated into 100μl of M63B1–0.4% Glu in a 96-well PVC microtiter plate (Falcon). After 4h incubation at 37°C, bacterial cells were stained for 15 minutes at room temperature by adding 50 μl of 0.5% (wt/vol) aqueous solution of crystal violet in each well. After five washes with distilled water, the bound dye was released from stained cells using 95% ethanol and measured by absorbance at 570 nm.

Biofilm formation was performed in 60 ml aerated microfermentors to assess mature biofilm formation as described by Ghigo et *al*. [35]. Continuous flow of 100ml/h of M63B1–0.4% Glu medium and constant aeration with sterile pressed air (0.3 bar) were used. After 24h of incubation, mature biofilms formed on the removable glass slide were dislodged by vortexing and sonication and resuspended in saline. Bacterial biomass was quantified by determining the number of CFUs.

### Cell line growth and adhesion assays

Intestine 407 (Int-407) cells derived from human embryonic intestinal epithelium were purchased from American Type Culture Collection (ATCC strain CCL-6). Cells were grown in a humidified incubator at 37°C under 5% CO_2_. Cells were cultured in Dulbecco’s Modifed Eagle Medium (DMEM) (PAA-laboratories GmbH—Dominique Dutscher) containing 10% (v/v) heat-inactivated fetal bovine serum (PAA-laboratories GmbH—Dominique Dutscher), 50 U/mL penicillin, and 50 μg/mL streptomycin. Adhesion assays were conducted in biological and technical triplicate over three to five successive passages of Int-407 cells. Briefly, monolayers were seeded with 4 × 10^5^ cells per well in 24-well tissue culture plates (Polylabo, Strasbourg, France) and incubated for 24 h. Monolayers were then infected at a multiplicity of infection (MOI) of 100 bacteria per cell in 1 ml of the cell culture medium without antibiotics and with heat-inactivated fetal calf serum (FCS) (PAA-laboratories GmbH—Dominique Dutscher). After incubation for 4h at 37°C in an atmosphere of 5% CO_2_, unattached bacteria were removed by washing the cell monolayers four times with sterile PBS. After detachment of the cells by addition of 1mL of Triton 1% (in PBS) per well, the suspension was transferred into a 1.5 mL reaction tube and the number of adhering bacteria was determined by quantification of CFUs by plating dilution onto LB agar plates.

### *In vitro* plant adhesion assays

Seeds of wild type *Arabidopsis thaliana* (Col-O) were obtained from the Nottingham *Arabidopsis* Stock Center (NASC). For *in vitro* cultures, seeds were sterilized in 70% EtOH with 0.05% SDS followed by washing in 95% EtOH, dried and sown on germination medium containing 0.8% w/v agar, 1% w/v sucrose and half-strength Murashige & Skoog salts (M0255; Duchefa Biochemie, Netherlands). After 2 days of stratification at 4°C in darkness, plants were grown under long-day conditions (6h light/8h dark cycles) at 23°C. Fifteen-day-old *Arabidopsis* plants were used for the *in vitro* adhesion assays.

Bacterial adhesion was tested as described in Haahtela *et al*. [36]. Briefly, 2.5 x 10^6^ bacteria were incubated with whole plants in 5 ml of PBS at room temperature in a controlled environment incubator shaker set at 30 rpm for 4 hours. A non-infected plant control was maintained under the same conditions. The plants were then washed twice for 15 min with 10mL of saline (0.9 [w/s] sodium chloride) and homogenized in 1mL of saline with a tissue grinder (Kontes, size C), and the suspension was serially diluted and the number of adhering bacteria was determined by quantification of CFUs by plating the dilution onto LB agar plates. Each experiment was conducted in biological and technical triplicate.

### Mice Intestinal colonisation

Female specific-pathogen mice (OF1 Swiss; 3 to 5 weeks old, 22g, Charles River Swiss) were used. The models and protocols used in this study were all approved by the ethics committee of Auvergne (Comité Régional d'Ethique en Matière d'Expérimentation Animale Auvergne, CEMEEA C2EA-02) in compliance with the European Community guiding in the care and use of laboratory animals (86/609/CEE). A total of 50 animals were used in this experiment. Animals were housed five to a cage in a temperature-controlled room with a 12 h light/12 h dark cycle and were fed with Rodent Diet (A04-Safe, Epinay/Orge, France) ad libitum throughout the experiments. Cages were cleaned each two days. One day prior to infection, water was withdrawn and replaced with sterile water containing 5g of streptomycin per liter throughout the experiment. After 1 week of acclimation, 200 μl of bacterial suspension in sterile water (10^7^ CFU) were given intragastrically to each mouse. For competition assays, each of the 8 LM21Δ*usher*-*kana-strepto* mutants was mixed with wild type strain LM21 in equal amounts in 200μl sterile water and administered intragastrically to 5 mice per group. The mutant strains showing a deficiency in colonization were then tested individually (5 mice per strain) and in competition with their respective trans-complemented strain by the same procedure. After 1 day and subsequently every day for 10 days, feces were collected and homogenized in 1mL saline, and serial dilutions were plated onto selective media. As described above, the removed feces were plated onto streptomycin-containing LB plates to measure the total number of CFUs and onto streptomycin-kanamycin-containing LB plates to measure the number of Δ*usher* mutant CFUs. From these numbers, the exact ratio of mutant to wild type or transcomplement was calculated for inoculum and feces contents. Mice were euthanized by cervical dislocation on day 12. Each experiment was conducted in biological and technical triplicate.

### Western blot analysis of type 3 fimbriae expression

Overnight grown bacteria (8.10^9^) were mechanically disrupted by sonication in 2mL of 40mM Tris-buffer, and lysates were centrifuged for 10 min at 20,000 g to pellet unbroken cells. Supernatants were ultracentrifuged for 1h at 100,000g, and pellets suspended in 0.1 mL of sarkosyl 0.5% were then incubated for 30 min on ice to solubilize inner membrane. Samples were then ultracentrifuged 1h at 100,000g, and pellets were suspended in 0.05 mL of tris-buffer. Protein quantification was performed using Bradford Protein Assay (Biorad) reagent according the manufacturers’ recommendations. Five μg of protein samples were then analyzed by western blotting using an anti-MrkA polyclonal antibody (generous gift from Steven Clegg).

### Statistical analysis

For analysis of the significance of differences, Student’s t-test was used to compare data from the two groups. All experiments were made at least three times. A P-value of ≤ 0.05 was considered to be statistically significant. RT-PCR statistical analysis was performed using the non-parametric One-way ANOVA. P values of ≤ 0.05 were considered to be statistically significant.

### Accession Numbers

The INSDC accession numbers of usher proteins used in this study are listed below. *Klebsiella pneumoniae* HS11286 (AEW59011, AEW59085, AEW60001, AEW61304, AEW6304); *Klebsiella pneumoniae* MGH 78578 (ABR75716, ABR75717, ABR75730, ABR75944, ABR77102, ABR78394,ABR78675, ABR78797, ABR79828, ABR79894); *Klebsiella pneumoniae* NTUH-K2044 (BAH61229, BAH61295,BAH61460, BAH62173, BAH63378, BAH64776, BAH64779, BAH65059, BAH65073); *Klebsiella variicola* 342 (initially identified as *K*. *pneumoniae*: ACI08730, ACI09976, ACI10676, ACI09288, ACI10013, ACI08659, ACI06599, ACI08146, ACI08479); *Klebsiella pneumoniae* KCTC 2242 (AEJ96010, AEJ96079, AEJ96970, AEJ98151, AEJ99466, AEJ99752, AEJ99817, AEJ99818, AEJ99901); Klebsiella *pneumoniae* JM45 (AGT22732, AGT22745, AGT23011, AGT24313, AGT25490, AGT25491, AGT26343, AGT26409); *Klebsiella pneumoniae* CG43 (AGX39043, AGX39045, AGX41005, AGX40456); *Klebsiella pneumoniae* 1084 (AFQ64205, AFQ67915, AFQ67856, AFQ67011, AFQ65856, AFQ67240, AFQ64480, AFQ64205); *Klebsiella pneumoniae* Kp13 (AHE42797, AHE42813,AHE43103, AHE43106, AHE44628, AHE45921, AHE46162, AHE46764, AHE46837); *Klebsiella pneumoniae* 30684/NJST258_2 (AHM77757, AHM77758, AHM77775, AHM78064, AHM78067, AHM79571, AHM80854, AHM80923, AHM81143, AHM81806, AHM81807, AHM81882); *Klebsiella pneumoniae* 30660/NJST258_1 (AHM83349, AHM83350, AHM83368, AHM83664, AHM83667, AHM85224, AHM86458, AHM86526, AHM86817, AHM87489, AHM87490, AHM87517); *Klebsiella pneumoniae* LM21 (KPLM21_90123, KPLM21_160040, KPLM21_610081, KPLM21_1040039, KPLM21_200008, KPLM21 _1000123, KPLM21_90135, KPLM21_220012).

## Results

### Identification and characterization of CU fimbrial loci in *K*. *pneumoniae* LM21 genome

**Fig 1:**
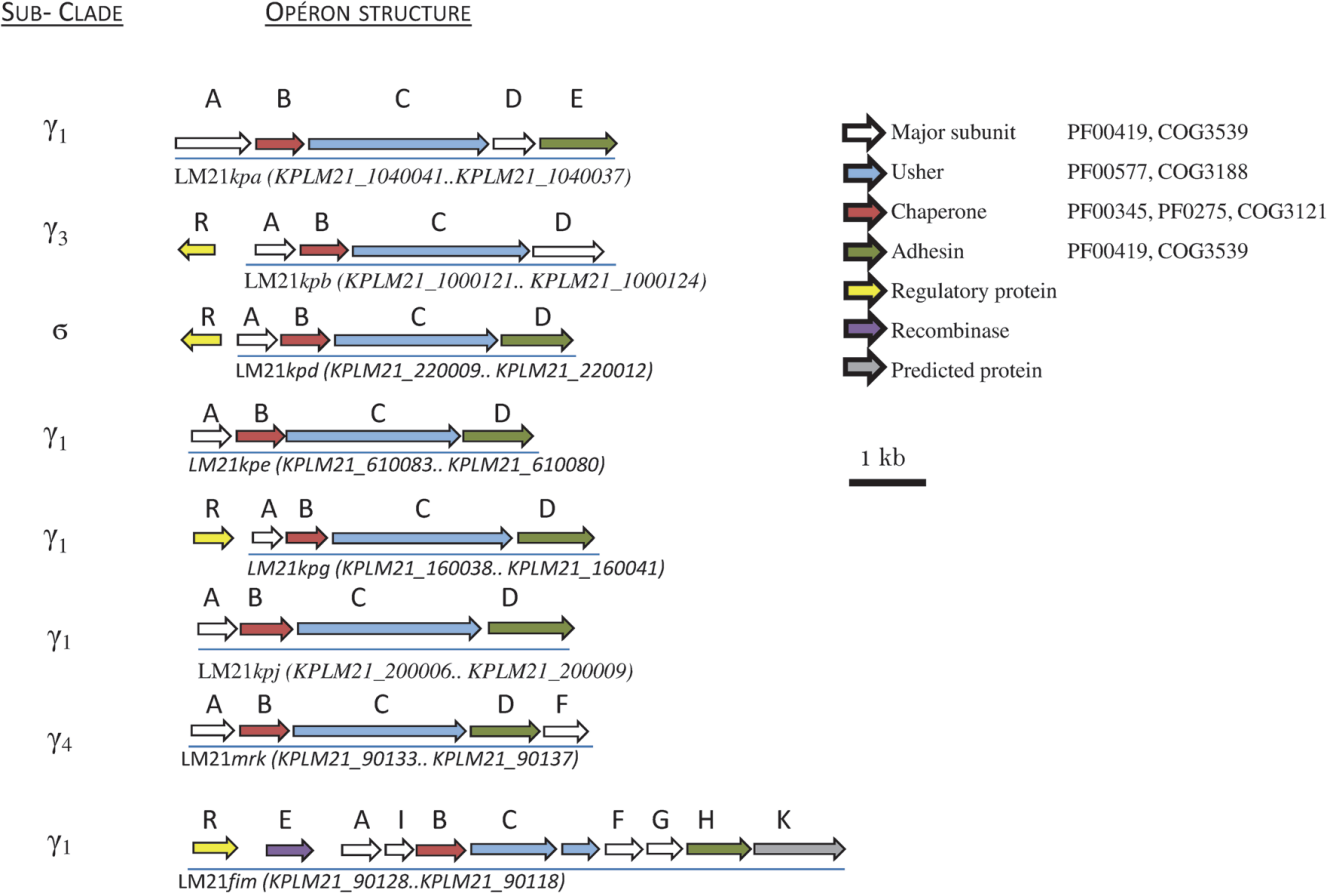
Genetic organization of CU fimbrial types identified in *Klebsiella pneumoniae* LM21. The genetic organization of the different fimbrial types is depicted diagrammatically. The designation of putative fimbrial genes and the locus tag of ORFs annotated in the *K*. *pneumoniae* LM21 genome are indicated. A total of eight fimbrial gene clusters and genes encoding putative regulators are shown. Each of these fimbrial loci is underlined. Fimbriae are grouped according to the Nuccio cladding scheme (Nuccio and Baümler, 2007). Genes are color-coded according to predicted function of the corresponding protein product, with associated Pfam and COG domains indicated (CGO and PF). The scale represents DNA length in kilo base pair. Reference locus tags for individual fimbrial types are displayed under the locus.

**Fig 2:**
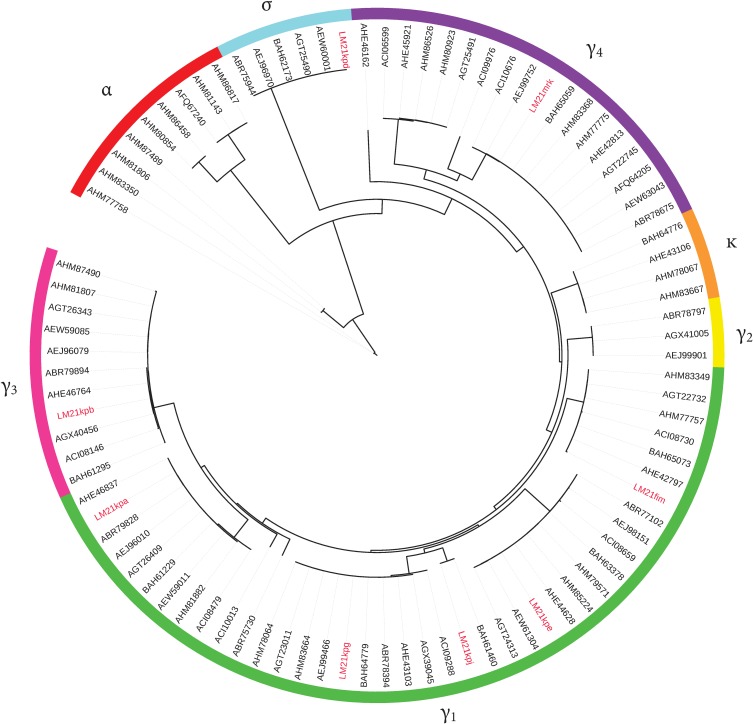
Circular phylogram of fimbrial usher proteins identified in *K*. *pneumoniae*. A total of 90 amino acid sequences deduced from the 7 to 9 CU loci of the twelve *K*. *pneumoniae* genomes available in the NCBI data bank were used to infer the evolutionary relationship of usher protein. Fimbrial gene clusters were grouped according to the Nuccio subclade system (α, β, ϭ, п, κ, γ) and highlighted in color. *K pneumoniae* LM21 usher proteins are leaf labeled in red.

In the *K*. *pneumoniae* LM21 genome, a total of eight potential CU fimbrial operons defined as polycistronic gene clusters containing at least one usher and one chaperone encoding sequence and flanked by one or more genes encoding fimbrial subunits were identified. The genetic organization of these CU fimbrial gene clusters (Fig. 1) was predicted by inspecting individual genes for conserved fimbrial protein domains. The usher proteins are members of a classical chaperone/usher family and share conserved domains (PFAM00577 and/or COG3188). The presence of usher encoding genes was confirmed by PCR using primers P1 to P16 (Table 1). The LM21 *K*. *pneumoniae* CU loci comprised type 1 and type 3 fimbrial gene clusters *fim* and *mrk* that are divergently clustered and transcribed. Five of the other six LM21 CU loci showed a strong similarity toward operons found out by Wu *et al*. [18] in strain NTUH-K2044. Together with type 1 and type 3, they showed a minimum of 93% of identity (coverage length >98%) when compared to NTUH-K2044 operon subunit, with an identity value between 95 and 100% for usher encoding genes. The common CU operons between LM21 and NTUH-K2044 strains were *kpa*, *kpb*, *kpd*, *kpe*, *kpg*, *mrk* and *fim* accordingly to the nomenclature proposed by Wu *et al*. [37]._._ The eighth LM21 CU operon detected, which corresponds to ORFs KPLM21_200008.KPLM21_200011, has not been described so far; as *kph* and *kpi* were proposed recently for novel CU clusters in *K*. *pneumoniae* strains BJ1-GA and SA1 [38], the novel cluster from LM21 was called *kpj*. Using these data as well as the sequences of the CU fimbriae usher detected in the twelve NCBI sequenced *K*. *pneumoniae* genomes (S1), which contain an average of 9 CU operons/genome (minimum 5 and maximum 12), a circular phylogram was constructed to display the evolutionary relationship of these amino acid sequences (See S1 Table). The circular phylogram of *K*. *pneumoniae* usher sequences demonstrated that the *K*. *pneumoniae* species contains representatives of the six clades defined by Nuccio & Baumler [3] (Fig. 2). The γ clade was the largest and encompassed 50 CU fimbrial types across five subclades.

The classification of the fimbrial gene clusters into CU clades based on the usher sequences was previously shown to correlate with the gene arrangement within clusters [39]. The eight LM21 usher loci belong to γκп (for seven of them) and ϭ clade (one of them, LM21*kpd*) (Fig. 1) and are characterized by a common pilus subunit homology domain (PFAM00419). Seven CU loci share the same gene organization encoding first the major subunit then a chaperone followed by an usher (MCU organization: M for major subunit, C for chaperone and U for usher) (Fig. 1). They are split into different subclades: LM21*kpa*, LM21*kpe*, LM21*kpg*, LM21*kpj and* LM21*fim* are MCUT (T for Tip adhesin)-organized and belong to γ1 subclade. They share, like all γ clade members, the PFAM00419 subunit domain and a COG3539 domain, which are also found in subunits of γ1 and γ2 fimbriae. The LM21*mrk* operon, which is MCUT-organized, belongs to the γ_4_—fimbriae. Unlike other members of the γ-fimbriae, the LM21*kpb* operon belongs to the γ3-fimbriae with a MCUM (M for Major subunit) organization that does not contain the conserved domains PFAM00419 and COG3539 but PFAM04619 subunit domain and PFAM02753 chaperone domain (Fig. 1). Finally, the LM21*kpd* operon belongs to the ϭ cluster with an MCUT core operon structure and has significant homology to COG5430 domains in its subunits. Chaperones of the LM21*kpd* operon contain either a COG00345 domain only or a COG3121 domain and a PFAM00345 domain. No tip adhesin was present in this locus.

Real time RT-PCR analysis performed using RNA samples from bacteria grown in rich (LB, DMEM) or minimal (M63B1) media used for phenotypical characterization showed that the eight LM21 usher encoding genes were all expressed, whatever the growth conditions. Compared to the *rpoB* housekeeping gene, there was no variation of usher gene expression between the different media, excepted for *kpgC* which was expressed at higher level in DMEM compared to LB (p<0.05; ANOVA) (Table 3).

**Table 3:** RT-PCR analysis of usher genes expression in M63B1, LB and DMEM media.

	Culture medium		
target gene	M63B1	LB	DMEM
***kpaC***	46.6 ± 10.7	51.6 ± 8.2	49.6 ± 21.1
***kpbC***	7.1 ± 1.2	7.5 ± 0.8	7.1 ± 0.4
***kpdC***	52.0 ± 2.2	40.9 ± 3.2	55.1 ± 11.0
***kpeC***	441.3 ± 81.7	382.4 ± 83.1	344.4 ± 58.5
***kpgC***	93.6 ± 4.2	111.6 ± 20.4	75.3 ± 8.5
***kpjC***	94.6 ± 9.9	81.4 ± 20.3	92.6 ± 5.7
***mrkC***	16.6 ± 0.6	17.2 ± 2.9	14.7 ± 1.5
***fimC***	30.5 ± 1.4	34.1 ± 1.5	28.2 ± 4.9

Results were expressed in fold-decreased expression (±SD) compared to *rpoB* housekeeping gene expression level. Expression levels were compared by nonparametric one-way ANOVA, comparing expression of each usher gene in the three different media. Only *kpgC* expression was higher in DMEM compared to LB; p < 0.05 (ANOVA).

To investigate the role of the LM21 *K*. *pneumoniae* CU loci, isogenic deletion mutants were created by allelic replacement of each of the eight potential encoding usher genes. The growth rate of each mutant was similar to that of the wild type (S1 Fig.). Western blot analysis performed with MrkA specific antibodies showed that all usher deleted mutants, except Δ*mrkC*, expressed type 3 pili at their cell surface (S2 Fig.). In addition, assessment of the expression of the 7 usher genes in Δ*mrkC* mutant by RT-PCR indicated that there was no significant variation compared to the wild-type strain (S2 Table).

### Adhesion and colonization phenotype of usher deletion mutants

**Fig 3:**
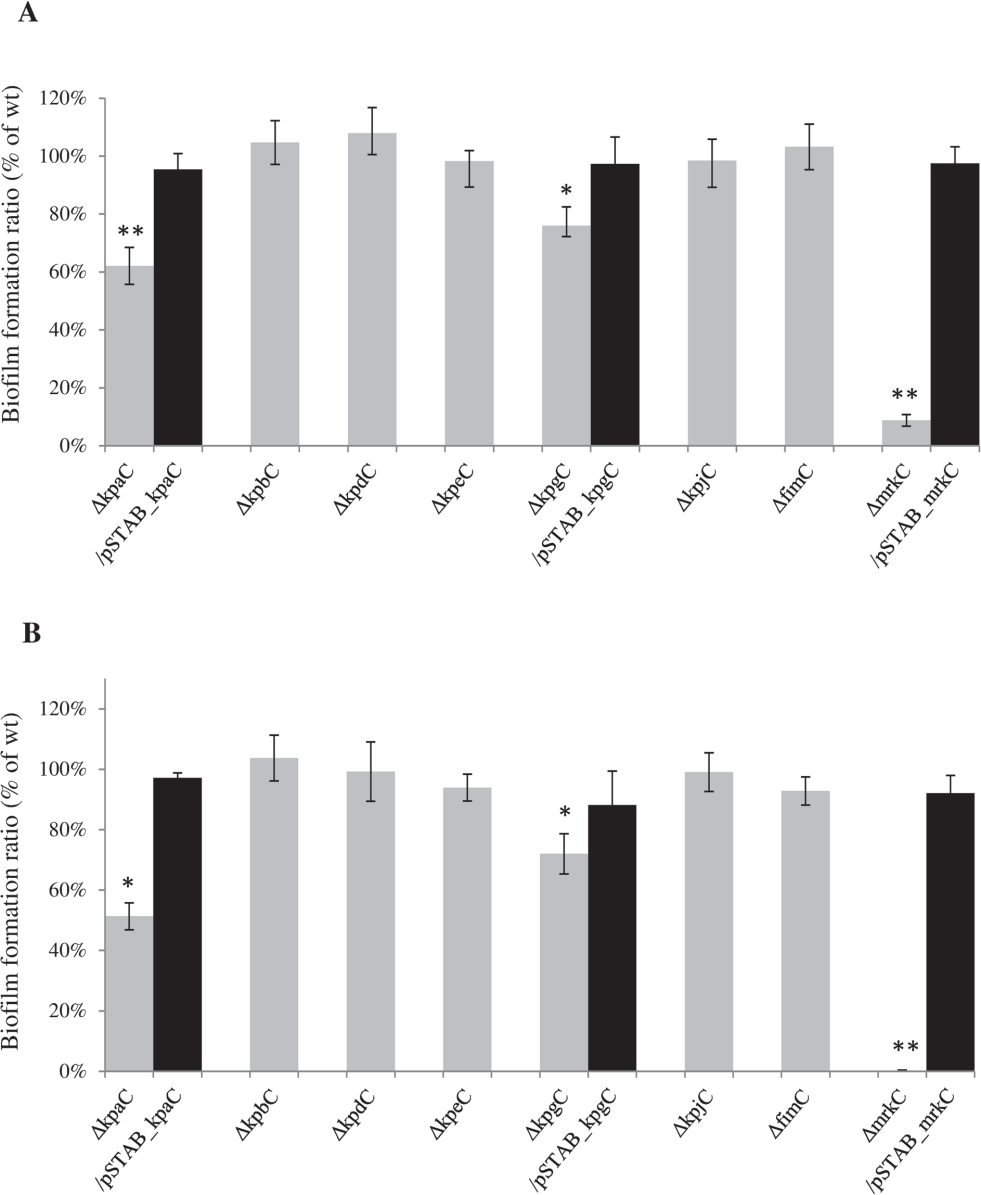
Biofilm formation capacity of the *K*. *pneumoniae* LM21*Δusher* mutants strains and, for three of them, their transcomplemented strains. Biofilms developed were quantified (A) by crystal violet staining on microtiter plates after 4 hours of incubation and (B) by CFU determination after 24 hours of incubation in the microfermentor model, as described in experimental procedures. Data are means of measurement made in triplicate. The biofilm formation ability of the mutant strains is expressed as a percentage of LM21 wild type biofilm, set to 100% (OD_600_ and CFU values for the *K*. *pneumoniae* LM21 wild type are respectively 0.52 and 1.24x10^9^). The error bars represent standard errors of the means. Significant differences are indicated by * and ** for p < 0.05 and p<0.01, respectively (Student’s t-test).

Determination of biofilm biomass using CV staining in microtiter plates and biomass determination in the microfermentor device indicated that three mutants (LM21Δ*kpaC*, LM21Δ*kpgC* and LM21Δ*mrkC*) were impaired in their biofilm formation ability compared to the wild type *K*. *pneumoniae* LM21 strain (Fig. 3). The ability to form biofilm was partially restored by transcomplementation with the wild type usher encoding gene (up to 80% of the wild type level), whatever the biofilm formation model used (Fig. 3A and 3B).

**Fig 4:**
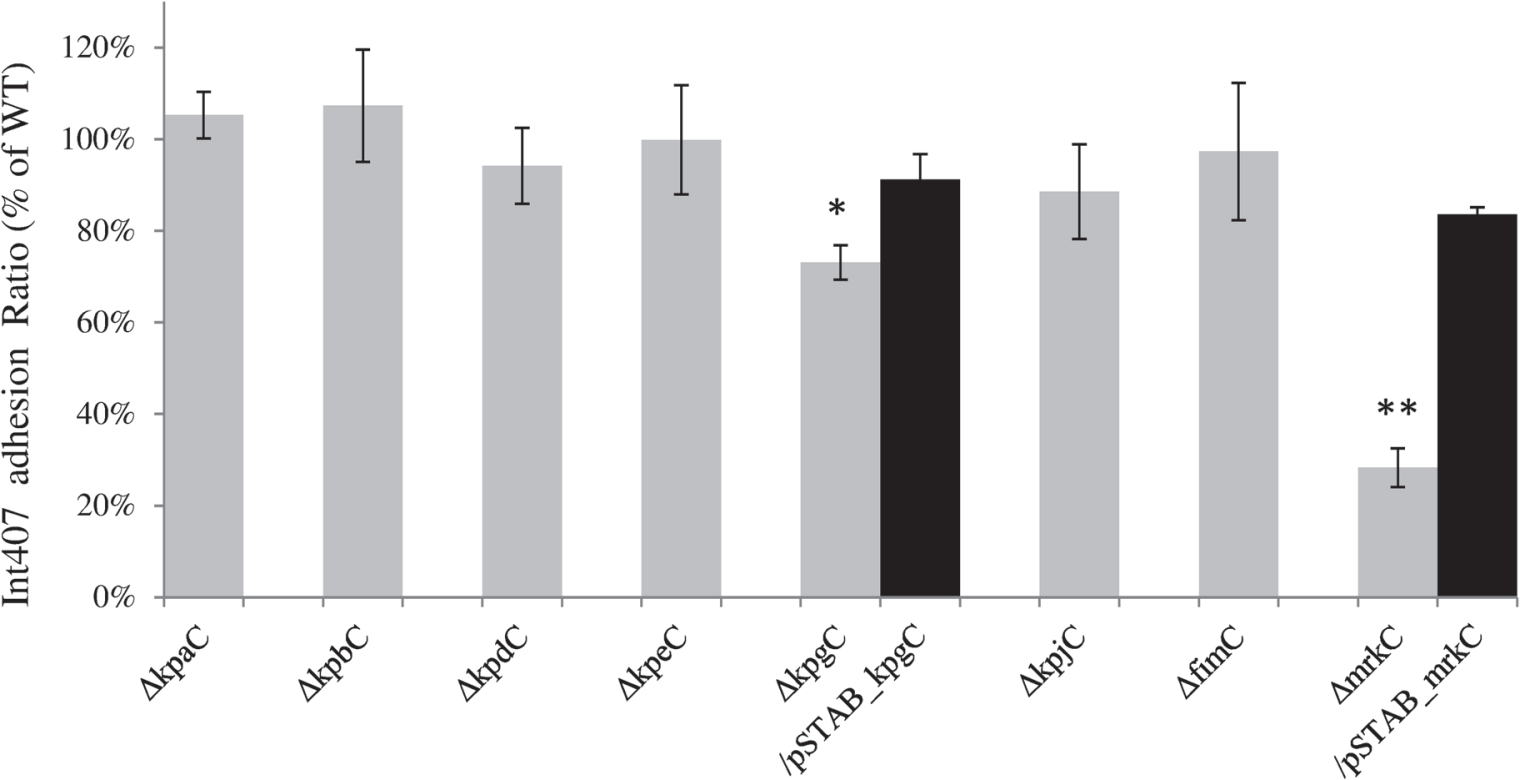
Adhesion assays to Int-407 cells of the LM21*Δusher* mutants strains and, ² for two of them, their transcomplemented mutants. Results are expressed as the percentages of LM21 wild type adhesion, set to 100% (CFU value for *K*. *pneumoniae* LM21 wild type was 1.93x10^9^). Data are the means of measurements made in biological and technical triplicate. Significant differences are indicated by * p < 0.05 and ** for p<0.01 (Student’s t-test).

The eight usher-deleted mutants were also tested for their ability to adhere to human intestinal Int-407 cells. Two of the mutants (LM21Δ*kpgC* and LM21Δ*mrkC*) adhered less than the wild type strain LM21, (75% and 20%, respectively) whereas the six other mutants did not show any difference compared to the parental strain. The adhesion phenotype was partially restored by the trans-complemented strains (up to 80% of the wild type level) (Fig. 4).

**Fig 5:**
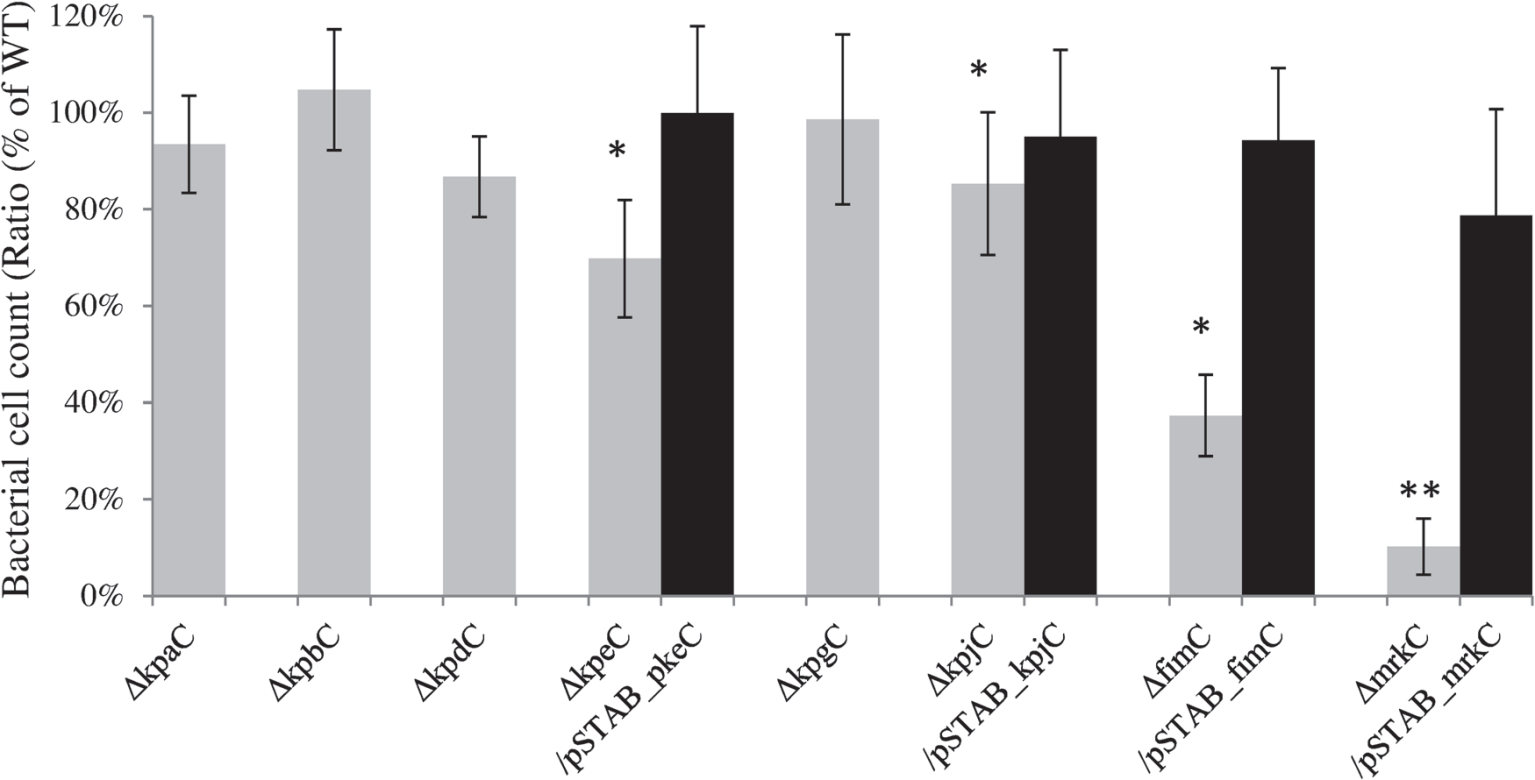
Adhesion assays to *Arabidopsis thaliana* whole seedlings of the *K*. *pneumoniae* LM21*Δusher* mutants strains and, for four of them, their transcomplemented mutants. Results are expressed as the percentages of LM21 wild type adhesion, set to 100% (CFU value for *K*. *pneumoniae* LM21 wild type was 1.47.10^7^). Data are the means of measurements made in biological and technical triplicate. Significant differences are indicated by * and ** for p < 0.05 and p<0.01 respectively (Student’s t-test).

Adhesion assays performed with whole 15-day-old *A*. *thaliana* specimens showed that four mutants, LM21Δ*kpeC*, LM21Δ*kpjC*, LM21Δ*fimC* and LM21Δ*mrkC*, adhered at significantly lower levels than those of the wild type strain (P<0.01 with LM21Δ*kpjC* and P<0.05 for LM21Δ*kpeC*, LM21Δ*fimC)* (Fig. 5).

**Fig 6:**
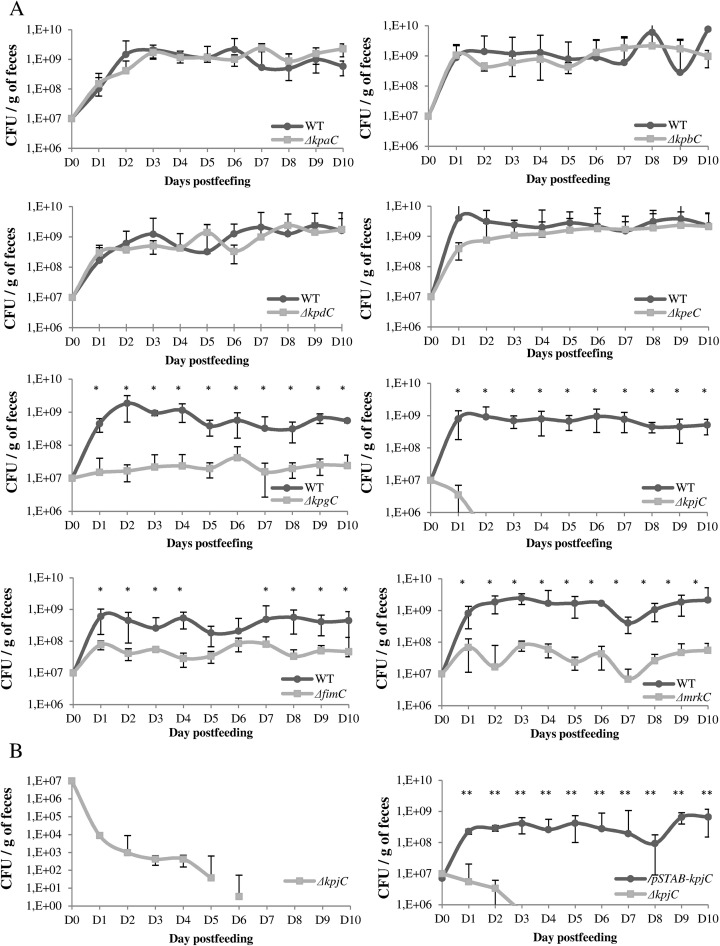
Colonization assays in the murine model of *K. pneumoniae* LM21*Δusher* mutants strains and trans-complemented mutants. (A) The colonization properties of the strains are shown as the competition between the wild type and the eight isogenic mutants. (B) For mutants showing an highly attenuated phenotype, individual assays involving the mutant alone and in competition assays with its trans-complemented strain were conducted. Data are means of measurement made with 5 mice per group. Significant differences are indicated by * and ** for p < 0.05 and p<0.01, respectively (Student’s t-test).

Determination of the ability of each usher mutant to colonize mice intestinal tract concurrently with the parental wild type strain indicated that four mutants, LM21Δ*kpgC*, LM21Δ*kpjC*, LM21Δ*fimC* and LM21Δ*mrkC*, were significantly impaired compared to the levels of the wild type strain (Fig. 6A) (P<0.01 by the student’s test for LM21Δ*kpgC and* LM21*mrkC* P<0.05 for *fimC* and P<0.01 for LM21Δ*kpjC*). Further colonization assays performed with the highest attenuated mutant, LM21Δ*kpjC*, showed that this mutant alone was also unable to colonize the intestinal tract and was outcompeted by its trans-complemented LM21Δ*kpjC/*pSTAB-*kpjC* mutant (Fig. 6B).

## Discussion

To detect CU clusters, the choice of the usher as target gene was conditioned by the fact that one usher encoding gene is ubiquitously associated with all CU gene clusters and is present in a single copy in all CU gene clusters so far described. A total of eight potential usher-encoding genes were detected in *K*. *pneumoniae* LM21 genome, all of them harboring adjacent cognate chaperone-encoding and fimbrial subunit-encoding genes. Whereas genes for CU pathways have been shown to be encoded on both chromosomal and plasmid locations [40,41], the eight CU loci detected in the genome of *K*. *pneumoniae* LM21 were located on the chromosome and most of them belonged to the γ_1_ subclade. This result highlights the main role of this subclade in bacterial survival and pathogeny processes as previously suggested [41]. In addition, analysis of the surrounding regions of each CU operon did not reveal potential mobile DNA sequences (data not shown), suggesting that these operons have not been acquired through recent lateral gene transfers [42].

Of the eight CU-loci detected in *K*. *pneumoniae* LM21 genome, two corresponded to previously well-characterized CU operons. The locus with ORFs KPLM21_90133 to KPLM21_90137 was identified as the type 3-encoding *mrk* operon previously described in all members of the family *Enterobacteriaceae*. While first identified and characterized in *Klebsiella*, type 3 pili are commonly found in other *Enterobacteriaceae*, and *mrk* gene clusters are mostly chromosome-borne [7,43,44]. m*rk* operon belongs to γ_4_ clade and shares the core operon structure present in all members of the γ_4_ clade [3]. The type 3 fimbriae are characterized by their ability to agglutinate erythrocytes treated with tannic acid *in vitro*, and this phenotype has been referred to as the mannose-resistant *Klebsiella-*like hemagglutination (MR/K) reaction [45]. Type 3 fimbriae have also been shown to mediate attachment to endothelial and bladder epithelial cell lines and to play a role in biofilm formation on abiotic surfaces and surfaces coated with host-derived materials [2,46–51]. Previous studies have also reported that type 3 fimbriae are efficient in promoting enterobacterial adherence to the roots of various grasses and cereals [36]. In our study, deletion of the usher-encoding ORF of this locus impaired all tested phenotypes, including murine intestinal colonization contrary to the results obtained after deletion of the whole *mrk* operon by Struve *et al*. [47]. It has been previously demonstrated that type 3 fimbriae deletion down-regulates type 1 fimbrial expression [2]. In this study, deletion of *mrkC* did not significantly modify the expression of the 7 other usher genes in strain LM21 (S2 Table), indicating no cross-regulation. Altogether, these results suggest that LM21 type 3 pili is a major actor in bacterial adherence both *in vitro* and *in vivo* by means of a large array of targets and tissue tropisms.

Type 1 fimbriae-encoding operon was the second previously well-known CU operon detected in our study. These fimbriae are found in virtually all members of the family *Enterobacteriaceae* [12]. The genetic organization of the *K*. *pneumoniae fim* operon resembles that of *E*. *coli fim* and contains homologs of all nine *fim* genes described previously [3,37]. *fimC* gene knock-out experiment in *K*. *pneumoniae* LM21 did not impair its biofilm formation capacity, as previously described for other *K*. *pneumoniae* strains [51–54]. However, these results are at variance with those obtained in the closely model *E*. *coli*, in which type 1 fimbriae have been shown to promote biofilm formation [53]. This intriguing difference could be related to the characteristic production of copious amounts of capsular material by *K*. *pneumoniae* that impedes type 1 functionality during biofilm formation due to the shortness of these pili [55,56].

In agreement with van Aarsten et *al*.,[57], we observed no significant difference between the LM21Δ*fimC* mutant and its parental strains in the *in vitro* animal cell adhesion assay. However, this mutant was impaired in its ability to adhere to *Arabidopsis* seedlings (Fig. 5), suggesting that the type 1 fimbriae are involved in *K*. *pneumoniae* plant adhesion, as in the grass model of Haahtela *et al*. [36]. In addition, and in contrast with previous reports [54,57], murine intestinal co-colonization assay performed with LM21Δ*fimC* mutant showed that the mutant had a lower colonization capacity than the wild type.

In addition to type 3 and type 1-encoding operons, *K*. *pneumoniae* LM21 genome harbored six other CU fimbriae systems. The newly identified *kpj* cluster was shown to be involved in adhesion to *Arabidopsis* tissues and in murine colonization. Determination of the ability of each mutant to colonize the murine intestinal tract was initially performed in co-colonization assays including the wild-type strain. To avoid misinterpretation due to concurrent colonization processes that potentially influence the host immune status and thus modify the capacity of both microorganisms to establish, the LM21Δ *kpj* mutant was assessed individually in the murine model; the bacterial load in the animals feces rapidly declined, indicating the mutant was also impaired in its colonization capacity when given alone (Fig. 6B). Interestingly, the LM21Δ*kpjC* mutant was not impaired in its capacity to adhere to Int-407 cells, suggesting the adhesin potentially encoded by this operon does not recognize receptors at the surface of intestinal cells but rather interacts with other gastro-intestinal components.

Deletion of the LM21*kpjC* usher-encoding gene significantly decreased its ability to form biofilm and its ability to adhere *in vitro* to Int-407 and plant cells, whereas deletion of LM21*kpaC* usher gene was only associated with a decreased ability to form biofilm in both early and late stages. These results suggest that the different fimbrial adhesins harbored by *K*. *pneumoniae* have specific role allowing the bacteria to adhere to different receptors present in different niche and environnement. It has previously been demonstrated that type 1 fimbrial expression is up-regulated in wild type *K*. *pneumoniae* infecting the bladder, but is down-regulated in cells colonizing the intestinal tract or infecting the lungs [54].

Regarding the loci LM21*kpb*, LM21*kpd* and LM21*kpe*, no clear *in vitro* or *in vivo* role was identified whatever the phenotype investigated, except for LM21*kpe*, for which deletion induced a slightly decreased adhesion in the plant model. We demonstrated in this study that the 8 LM21 CU usher genes were expressed *in vitro* even though specific demonstration of the presence of corresponding fimbriae at the bacterial cell surface still requires further investigations. Numerous cryptic fimbrial operons have been detected in the genomes of *E*. *coli* K-12, *E*. *coli* O157:H7 and *Salmonella*, whose expression is subject to phase variation in response to environment cues [58–60]. Each bacterium likely expresses a few pili type at a given time according to its growth or virulence requirements. Besides, by analogy with other CU systems, the upregulation of expression and biosynthesis of fimbriae could involve a complex interplay of multiple transcriptional regulator, invertible promoter switchs or DNA methylation-based systems, in addition to a potential regulation by the levels of expression of other surface components [5,61–63].

In conclusion, we report the characterization of eight CU loci on the genome of *K*. *pneumoniae* LM21. Using several *in vitro* and *in vivo* experimental models, we were able to show the involvement of five of them in bacterial adhesion or colonization processes, demonstrating therefore the large adhesion capacity of this species. However, complete demonstration of its adhesion capacities still requires the identification of the substrates recognized by the potential adhesins harbored by the non-yet phenotypically characterized CU operons. In addition, epidemiological studies assessing the presence and the expression of these loci in a large panel of *K*. *pneumoniae* isolates from different sources will complete the elucidation of their respective role.

## Supporting Information

S1 Table Strains labels and accession numbers of gene encoding usher identified in *Klebsiella pneumoniae* strains genomes present in NCBI and in this study. (PDF)

S2 Table RT-PCR analysis of the expression of usher genes in the *mrkC*-deleted mutant (type-3 pili usher) and the LM21 wild-type strain. (PDF)

S1 Fig Growth curves of LM21 wild-type strain and its usher-deleted mutants. Bacterial cells were collected every 30 min for 8.5 hours and plated on media. Results are expressed as the number of CFU/ml.
(TIF)

S2 Fig Western blot of bacterial surface extracts of the usher-deleted mutants and the LM21 wild-type strain. The figure shows an immunoblot of a gel on which 5 μg of extract from each bacterial strain has been loaded. The gel was immunostained with an antibody that recognizes the major subunit of type 3 pili (MrkA). MW, molecular weight size marker.
(TIF)
