# Supplementary material for: In Silico Analysis of Usher Encoding Genes in Klebsiella pneumoniae and Characterization of Their Role in Adhesion and Colonization
Source: PLoS One. 2015 Mar 9;10(3):e0116215. doi: 10.1371/journal.pone.0116215 (PMC4353729; doi:10.1371/journal.pone.0116215)
Supplement: S1 Table — (PDF) [file pone.0116215.s001.pdf]

Table S1. Strains labels and accession numbers of gene encoding usher identified in *Klebsiella pneumoniae* strains genomes present in NCBI and in this study

Fimbrial Clade	Usher Accession	Organism
κ	BAH64776	Klebsiella pneumoniae subsp. pneumoniae NTUH-K2044
κ	AHE43106	Klebsiella pneumoniae subsp. pneumoniae Kp13
κ	AHM78067	Klebsiella pneumoniae 30684/NJST258_2
κ	AHM83667	Klebsiella pneumoniae 30660/NJST258_1
γ1	AEW59011	Klebsiella pneumoniae subsp. pneumoniae HS11286
γ1	AEW61304	Klebsiella pneumoniae subsp. pneumoniae HS11286
γ1	ABR75730	Klebsiella pneumoniae subsp. pneumoniae MGH 78578
γ1	ABR77102	Klebsiella pneumoniae subsp. pneumoniae MGH 78578
γ1	ABR78394	Klebsiella pneumoniae subsp. pneumoniae MGH 78578
γ1	ABR79828	Klebsiella pneumoniae subsp. pneumoniae MGH 78578
γ1	BAH61229	Klebsiella pneumoniae subsp. pneumoniae NTUH-K2044
γ1	BAH61460	Klebsiella pneumoniae subsp. pneumoniae NTUH-K2044
γ1	BAH63378	Klebsiella pneumoniae subsp. pneumoniae NTUH-K2044
γ1	BAH64779	Klebsiella pneumoniae subsp. pneumoniae NTUH-K2044
γ1	BAH65073	Klebsiella pneumoniae subsp. pneumoniae NTUH-K2044
γ1	ACI08730	Klebsiella pneumoniae 342
γ1	ACI09288	Klebsiella pneumoniae 342
γ1	ACI10013	Klebsiella pneumoniae 342
γ1	ACI08659	Klebsiella pneumoniae 342
γ1	ACI08479	Klebsiella pneumoniae 342
γ1	AEJ96010	Klebsiella pneumoniae KCTC 2242
γ1	AEJ98151	Klebsiella pneumoniae KCTC 2242
γ1	AEJ99466	Klebsiella pneumoniae KCTC 2242
γ1	AEJ99817	Klebsiella pneumoniae KCTC 2242
γ1	AEJ99818	Klebsiella pneumoniae KCTC 2242
γ1	AGT22732	Klebsiella pneumoniae JM45
γ1	AGT23011	Klebsiella pneumoniae JM45
γ1	AGT24313	Klebsiella pneumoniae JM45
γ1	AGT26409	Klebsiella pneumoniae JM45
γ1	AGX39045	Klebsiella pneumoniae CG43
γ1	AHE42797	Klebsiella pneumoniae CG43
γ1	AHE43103	Klebsiella pneumoniae subsp. pneumoniae Kp13
γ1	AHE44628	Klebsiella pneumoniae subsp. pneumoniae Kp13
γ1	AHE46837	Klebsiella pneumoniae subsp. pneumoniae Kp13
γ1	AHM77757	Klebsiella pneumoniae 30684/NJST258_2
γ1	AHM78064	Klebsiella pneumoniae 30684/NJST258_2
γ1	AHM79571	Klebsiella pneumoniae 30684/NJST258_2
γ1	AHM81882	Klebsiella pneumoniae 30684/NJST258_2
γ1	AHM83349	Klebsiella pneumoniae 30660/NJST258_1
γ1	AHM83664	Klebsiella pneumoniae 30660/NJST258_1
γ1	AHM85224	Klebsiella pneumoniae 30660/NJST258_1
γ1	AHM87517	Klebsiella pneumoniae 30660/NJST258_1
γ1	KPLM21_90123	Klebsiella pneumoniae 30660/NJST258_1
γ1	KPLM21_160040	Klebsiella pneumoniae LM21
γ1	KPLM21_610081	Klebsiella pneumoniae LM21
γ1	KPLM21_1040039	Klebsiella pneumoniae LM21
γ1	KPLM21_200008	Klebsiella pneumoniae LM21

γ2	ABR78797	Klebsiella pneumoniae subsp. pneumoniae MGH 78578
γ2	AEJ99901	Klebsiella pneumoniae KCTC 2242
γ2	AGX41005	Klebsiella pneumoniae CG43
γ3	AEW59085	Klebsiella pneumoniae subsp. pneumoniae HS11286
γ3	ABR79894	Klebsiella pneumoniae subsp. pneumoniae MGH 78578
γ3	BAH61295	Klebsiella pneumoniae subsp. pneumoniae NTUH-K2044
γ3	ACI08146	Klebsiella pneumoniae 342
γ3	AEJ96079	Klebsiella pneumoniae KCTC 2242
γ3	AGT26343	Klebsiella pneumoniae JM45
γ3	AGX40456	Klebsiella pneumoniae CG43
γ3	AHE46764	Klebsiella pneumoniae subsp. pneumoniae Kp13
γ3	AHM81807	Klebsiella pneumoniae 30684/NJST258_2
γ3	AHM87490	Klebsiella pneumoniae 30660/NJST258_1
γ3	KPLM21_1000123	Klebsiella pneumoniae LM21
γ4	AEW63043	Klebsiella pneumoniae subsp. pneumoniae HS11286
γ4	ABR78675	Klebsiella pneumoniae subsp. pneumoniae MGH 78578
γ4	BAH65059	Klebsiella pneumoniae subsp. pneumoniae NTUH-K2044
γ4	ACI09976	Klebsiella pneumoniae 342
γ4	ACI06599	Klebsiella pneumoniae 342
γ4	ACI10676	Klebsiella pneumoniae 343
γ4	AEJ99752	Klebsiella pneumoniae KCTC 2242
γ4	AFQ64205	Klebsiella pneumoniae subsp. pneumoniae 1084
γ4	AGT22745	Klebsiella pneumoniae JM45
γ4	AGT25491	Klebsiella pneumoniae JM45
γ4	AHE42813	Klebsiella pneumoniae subsp. pneumoniae Kp13
γ4	AHE45921	Klebsiella pneumoniae subsp. pneumoniae Kp13
γ4	AHE46162	Klebsiella pneumoniae subsp. pneumoniae Kp13
γ4	AHM77775	Klebsiella pneumoniae 30684/NJST258_2
γ4	AHM80923	Klebsiella pneumoniae 30684/NJST258_2
γ4	AHM83368	Klebsiella pneumoniae 30660/NJST258_1
γ4	AHM86526	Klebsiella pneumoniae 30660/NJST258_1
γ4	KPLM21_90135	Klebsiella pneumoniae LM21
σ	AEW60001	Klebsiella pneumoniae subsp. pneumoniae HS11286
σ	ABR75944	Klebsiella pneumoniae subsp. pneumoniae MGH 78578
σ	BAH62173	Klebsiella pneumoniae subsp. pneumoniae NTUH-K2044
σ	AEJ96970	Klebsiella pneumoniae KCTC 2242
σ	AFQ67240	Klebsiella pneumoniae subsp. pneumoniae 1084
σ	AGT25490	Klebsiella pneumoniae JM45
σ	AHM77758	Klebsiella pneumoniae 30684/NJST258_2
σ	AHM80854	Klebsiella pneumoniae 30684/NJST258_2
σ	AHM81143	Klebsiella pneumoniae 30684/NJST258_2
σ	AHM81806	Klebsiella pneumoniae 30684/NJST258_2
σ	AHM83350	Klebsiella pneumoniae 30660/NJST258_1
σ	AHM86458	Klebsiella pneumoniae 30660/NJST258_1
σ	AHM86817	Klebsiella pneumoniae 30660/NJST258_1
σ	AHM87489	Klebsiella pneumoniae LM21
σ	KPLM21_220012	Klebsiella pneumoniae LM21
