# Supplementary material for: In Silico Analysis of Usher Encoding Genes in Klebsiella pneumoniae and Characterization of Their Role in Adhesion and Colonization
Source: PLoS One. 2015 Mar 9;10(3):e0116215. doi: 10.1371/journal.pone.0116215 (PMC4353729; doi:10.1371/journal.pone.0116215)
Supplement: S2 Table — (PDF) [file pone.0116215.s002.pdf]

Table S2. RT-PCR analysis of usher genes expression in the Δ*mrkC* mutant. Results are expressed in fold change expression (±SD) in the Δ*mrkC* mutant compared to the wild-type strain. Expression levels were compared by nonparametric one-way ANOVA; $p > 0.05$ (ANOVA)

target genes	fold expression (±SD) compared to the wild-type strain
kpaC	1.6 ± 0.4
kpbC	2.3 ± 0.5
kpdC	2.1 ± 0.7
kpeC	1.8 ± 0.7
kpgC	1.3 ± 0.4
kpjC	1.9 ± 0.6
fimC	1.5 ± 0.7
