# Supplementary figures and images for: In Silico Analysis of Usher Encoding Genes in Klebsiella pneumoniae and Characterization of Their Role in Adhesion and Colonization
Source: PLoS One. 2015 Mar 9;10(3):e0116215. doi: 10.1371/journal.pone.0116215 (PMC4353729; doi:10.1371/journal.pone.0116215)

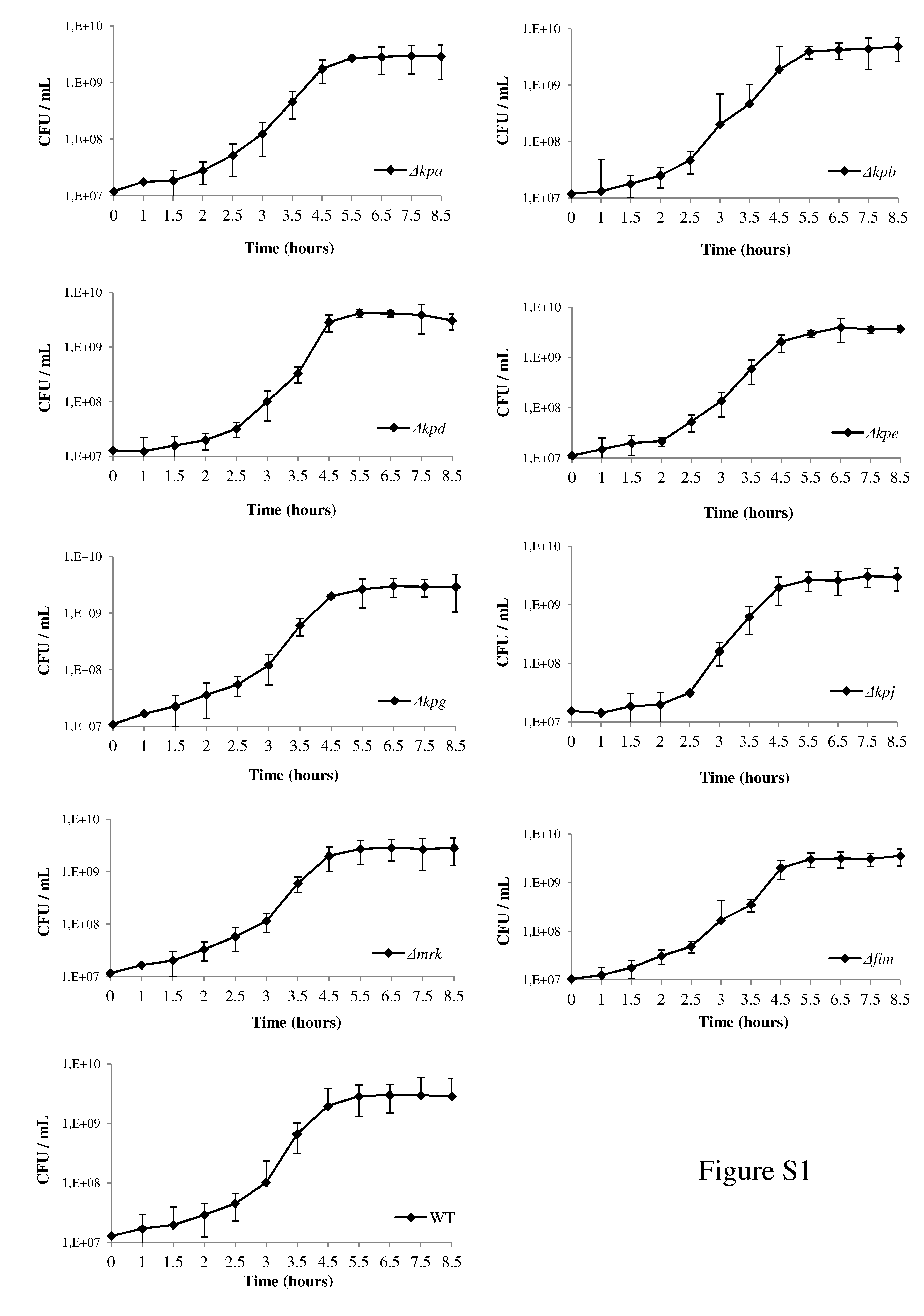

Supplement: S1 Fig — Bacterial cells were collected every 30 min for 8.5 hours and plated on media. Results are expressed as the number of CFU/ml. (TIF) [file pone.0116215.s003.tif]

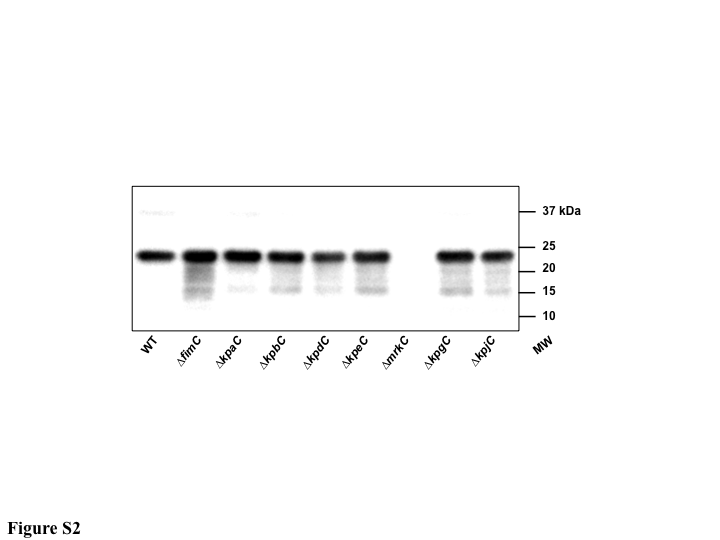

Supplement: S2 Fig — The figure shows an immunoblot of a gel on which 5 μg of extract from each bacterial strain has been loaded. The gel was immunostained with an antibody that recognizes the major subunit of type 3 pili (MrkA). MW, molecular weight size marker. (TIF) [file pone.0116215.s004.tif]
